# Extracorporeal Cardiopulmonary Resuscitation—Where Do We Currently Stand?

**DOI:** 10.3390/biomedicines13010204

**Published:** 2025-01-15

**Authors:** Brandon E. Ferrell, Jason Thomas, John P. Skendelas, Mayuko Uehara, Tadahisa Sugiura

**Affiliations:** 1 Montefiore Medical Center, Department of Cardiothoracic and Vascular Surgery, Bronx, NY 10467, USA; bferrell@montefiore.org (B.E.F.); jskendel@montefiore.org (J.P.S.); muehara@montefiore.org (M.U.); 2 Albert Einstein College of Medicine, Bronx, NY 10461, USA; jason.thomas@einsteinmed.edu

**Keywords:** extracorporeal cardiopulmonary resuscitation, cardiac arrest, extracorporeal membrane oxygenation, cardiopulmonary resuscitation, out-of-hospital cardiac arrest

## Abstract

Extracorporeal cardiopulmonary resuscitation (eCPR) is a method of acute resuscitation for patients who have suffered a cardiac arrest through the utilization of an extracorporeal membrane oxygenation (ECMO) pump. The use and efficacy of eCPR is an active area of investigation with ongoing clinical investigation across the world. Since its inception, ECMO has been utilized for several conditions, but more recently, its efficacy in maintaining cerebrovascular perfusion in eCPR has generated interest in more widespread utilization, particularly in cases of out-of-hospital cardiac arrest. However, successful implementation of eCPR can be technically challenging and resource intensive and has been countered with ethical challenges beyond the scope of conventional in-hospital ECMO care. The aim of this review is to summarize the status of eCPR in the current era.

## 1. Introduction

Cardiac arrest is defined as the complete loss of adequate perfusion to the body due to mechanical or obstructive failure. Sudden cardiac arrest is one of the leading causes of death worldwide, with approximately 356,461 people being treated for out-of-hospital cardiac arrest (OHCA) annually in the United States [1]. This is also a worldwide issue, with approximately an 8% survival rate for OHCA [2].

In the current healthcare environment, the American Heart Association (AHA) guidelines for the treatment of OHCA involve prompt activation of 9-1-1, bystander-initiated cardiopulmonary resuscitation (CPR), bystander and/or basic first responder application of an automated external defibrillator (AED), advanced cardiac life support (ACLS), and post-resuscitation care [1]. Although these interventions have maintained an acceptable survival rate, the overall prognosis remains poor, with greater than 70% of cases having resulted in unfavorable neurological conditions despite the return of spontaneous circulation (ROSC) [3].

Furthermore, post-cardiac arrest syndrome (PCAS) is a complex and multifaceted issue that often presents after successful resuscitation. Nolan et al. proposed this term for instances of acute brain injury, myocardial pathology, and systemic ischemic reperfusion response [4]. Myocardial damage occurs due to prolonged ischemic time experienced during a cardiac arrest. Ischemia–reperfusion injury (IRI) is another form of oxidative damage that is seen after ROSC when the return of blood flow over the transiently ischemic tissue results in the generation of toxic metabolites [5].

Various pharmacological and novel mechanical circulatory interventions have been developed to mitigate PCAS sequelae. One such method is initiation of venoarterial extracorporeal membrane oxygenation (VA ECMO) during CPR efforts. VA ECMO is an assistive device that simultaneously oxygenates and augments systemic blood flow [6]. Patients who have not regained ROSC with traditional ACLS can be placed on ECMO. Although the concept of eCPR is gaining interest, the use of assist devices for resuscitation in the field is not novel. A compact, portable, battery-operated pump was first developed at Baylor College of Medicine in 1976 for the treatment of moribund patients who could not be transported to the operating room, with as high as 38% survival at discharge [7]. The advancements of cardiopulmonary bypass, ECMO, and heparin-coated circuits have further helped in the widespread utilization of these devices for more than just OHCA [6]. Although widespread adoption of eCPR remains limited to select clinical sites, it remains a promising modality as mechanical circulatory technologies continue to improve [1,8].

## 2. Indications and Risk Factors

The use of eCPR in select patients with refractory cardiac arrest is currently a class 2a recommendation (level of evidence B-R) according to the 2023 American Heart Association updated ACLS guidelines [9]. The identification of patients that are (1) eligible for eCPR; (2) may achieve ROSC; and (3) may survive with acceptable neurologic outcomes are areas of active investigation. eCPR is typically only utilized after traditional ACLS has failed to achieve ROSC; however, various algorithms have been developed that can allow clinicians to predict the likelihood of successful eCPR interventions, which has resulted in variability across systems in terms of eligibility criteria [10].

One major indication for the usage of eCPR is if there is a reversible cause for the cardiac arrest. For instance, eCPR can be indicated for a classic case of a myocardial infarction (MI) followed by unsuccessful attempts at resuscitation. The use of ECMO can be considered as a bridge for coronary revascularization through percutaneous coronary intervention (PCI) or coronary artery bypass graft surgery (CABG). Extracorporeal support, in these cases, could allow for adequate end-organ perfusion to transport patients to a cardiac catheterization lab or operating room [11].

With few guidelines that remain in the field of eCPR, the French Ministry of Health published a self-made algorithm to determine the inclusion/exclusion criteria for patients to receive extracorporeal life support (ECLS). With these guidelines in place, the French government recorded survival as high as 20–30% with favorable neurological outcomes in patients treated with ECLS [12]. A comparative study consisting of 46 patients who underwent ECLS for shock (*n* = 25) or cardiac arrest (*n* = 21) found no statistical difference in survival on discharge between the ECLS use for cardiogenic shock vs. cardiac arrest (40% vs. 14%, *p* = 0.1) [13]. However, the only survivors from the cardiac arrest cohort were those who experienced an arrest in the catheterization laboratory or those who had accidental hypothermia.

Although there is no internationally accepted definition for the reversible causes of OHCA, it can broadly involve hypothermia, myocardial infarction and its sequelae, unstable arrhythmias, pulmonary embolism, and cardiotoxic drug overdose [14]. A systematic review by Debaty and colleagues showcased that out of the 841 eCPR-treated patients, a shockable rhythm was associated with twice the odds of favorable survival with good neurological outcomes [15]. Furthermore, the presence of a shockable rhythm contributes to the ‘Survival After Venoarterial ECMO’ (SAVE) score, developed by the Extracorporeal Life Support Organization (ELSO) based on data pooled from 160 U.S. and 120 international centers. The collaborative efforts of these centers allowed for the establishment of an online assessment tool for ECLS providers to assist in predicting survival for patients receiving ECMO for refractory cardiogenic shock [16,17]. However, the score does not currently stratify by ELCS in the setting of eCPR. More recently, the Prognostic Evaluation of ECPR (Pre-ECPR) score was proposed utilizing multivariable logistic regression and includes eight weighted predictors [18]. In internal cross-validation, it demonstrated more discriminatory performance than that of the ELSO criteria; however, it is a single-center study, and external validation is still needed [18].

Further indications for the usage of eCPR include a short no-flow (downtime) or low-flow (ACLS/time to cannulation) amongst patients. The current literature demonstrates an optimal no-flow time of <5 min and low-flow time to be <60 min [19,20]. While OHCA and in-hospital cardiac arrest (IHCA) are two different patient populations, two separate systematic reviews found that initial shockable rhythms, short low-flow time, and low lactate values at admission were predictors of better outcomes in each group [15,21]. Another retrospective study based on 500 adults who suffered an OHCA highlighted a positive neurological outcome with eCPR that was correlated with the presence of a witnessed arrest without asystole [22].

When considering someone for eCPR, age should also be considered. At the time of cannulation, consideration should be made if an older age patient is a candidate for advanced therapies (surgical or percutaneous interventions). If the patient is not, eCPR would likely be futile. Further, prior work from the University of Minnesota found a 30% decrease in survival with every 10-year increase in age (OR 0.70 (0.57–0.87), *p* = 0.001) in patients receiving ECPR for an OHCA with a shockable rhythm [23]. An analysis of the ELSO registry also found decreased hospital survival for patients > 65 years old undergoing eCPR despite controlling for comorbidities and illness severity [24].

Additional consideration needs to be given to the presence of active malignancy, severe peripheral vascular disease, known severe aortic insufficiency, aortic dissection, severe frailty, and pre-existing multiorgan dysfunction. The presence of any of these is a contraindication to eCPR.

## 3. Technical Considerations

There are several technical considerations one must consider when initiating eCPR. In terms of technique, a “percutaneous first” femoral approach is generally preferred. The femoral vessels are rapidly identified with anatomic landmarks (2–3 cm below the mid-inguinal point between the pubic tubercle and the anterior superior iliac spine) and/or an ultrasound device. If multiple team members are available, it is best to have one person obtain arterial access while another person works on venous access on the opposite side. Although femoral access is often selected for ease, jugular-femoral or jugular-axillary approaches can be used given adequate equipment and training. Surgical cannulation can also be utilized but is often reserved for cases in which percutaneous interventions have failed or patients with a history of extensive prior endovascular or surgical interventions involving the proposed access sites [25,26].

Cannula size should be chosen to allow for 2–2.5 L/m^2^/min of flow and is typically 14–18 French (arterial) and 19–25 French (venous) depending on body size [6]. When placing the venous cannula, visualization of the wire and cannula at the right atrial-inferior vena cava junction utilizing ultrasound is of the utmost importance to avoid misplacement of the cannula into the hepatic vein. When femoral arterial access is utilized, consideration of distal limb perfusion on the side of the arterial cannula is essential. In the setting of distal limb malperfusion (motor or sensory deficits, the lack of dopplerable pedal signals, or unilateral cooler limb), a 5–8 French distal perfusion cannula (DPC) should be placed in an antegrade (superficial femoral artery) or retrograde (posterior tibial or dorsalis pedis artery) manner and connected to the arterial limb of the ECMO circuit. Some institutions place a DPC catheter in all patients at the time of ECMO initiation.

After eCPR initiation, retrograde aortic flow is associated with increased afterload and left ventricular distension, particularly in patients with known aortic valve insufficiency. This increase in left ventricle pressure further increases the myocardial oxygen demand and pulmonary edema. Ventricular offloading can be accomplished with a combination of pharmacologic agents and mechanical devices, including an intra-aortic balloon pump (IABP), Impella (ABIOMED, Danvers, MA), or TandemHeart (LivaNova, London, UK). Other left ventricular unloading techniques include atrial septostomy to reduce left ventricular afterload [26]. Prior studies have shown improved hospital survival and successful weaning of patients who had LV offloading, especially when performed early (<12 h) [27,28].

## 4. Post-Arrest Care

**Figure 1:**
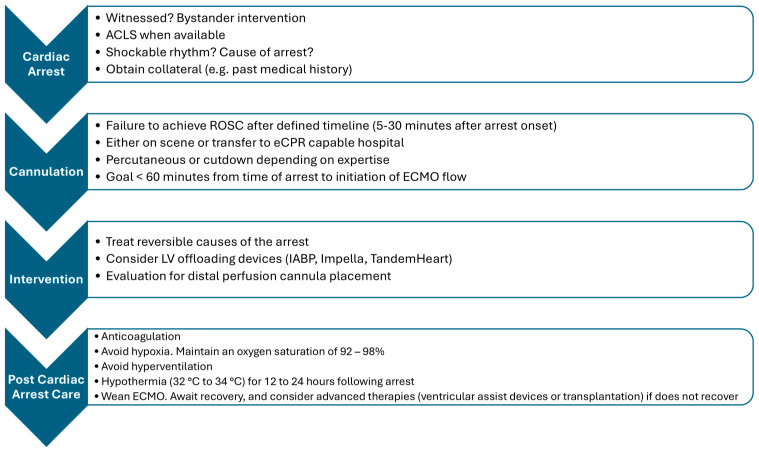
Key steps for the initiation and management of eCPR.

Post-cardiac arrest care (PCAC) involves immediate and intensive management of the airway and oxygenation, hemodynamics, and targeted temperature control (therapeutic hypothermia) (Figure 1). The principles of PCAC remain the same regardless of whether eCPR or traditional ACLS was utilized to achieve ROSC. A 12-lead ECG should be analyzed in all patients for the presence of ischemic changes, dysrhythmias, conduction defects, and low voltage (which may indicate tamponade). Abnormalities in the EKG may help to elicit the cause of the cardiac arrest and identify reversible pathology requiring emergent intervention. Furthermore, a portable chest X-ray (CXR) can be critical to visualize pulmonary etiologies such as edema, pneumothorax, and pneumonia. Correct positioning of the endotracheal tube can also be confirmed at this time. Bedside echocardiography should also be performed if there are suspicions regarding cardiac ischemia or tamponade. Global hypokinesis is typically anticipated; however, regional wall motion abnormalities may indicate ischemia. In patients suspected of having a pulmonary embolism (PE) who are unable to undergo a CT scan, echocardiographic signs of right ventricular dysfunction or, in some cases, direct visualization of the clot can help confirm the diagnosis. Additionally, pericardial tamponade and structural heart diseases, such as hypertrophic cardiomyopathy, can be quickly identified using bedside echocardiography [29].

According to PCAC guidelines, adequate oxygenation is of vital importance for good prognostic outcomes. The recommendation is to maintain a respiratory rate of 10–12 breaths per minute, along with the utilization of the lowest amount of oxygen required to maintain an oxygen saturation of 92–98% [30]. Though it is critical to prevent further hypoxemia to mitigate ongoing ischemic injury, hyperoxia poses risks as it may escalate oxidative stress and elevate free radical production, potentially resulting in tissue and neuronal damage. Current clinical investigations regarding the impact of hyperoxia on patient outcomes have produced inconsistent findings in terms of survival and neurologic function [31,32,33,34,35,36].

Hyperventilation should be avoided. It results in increased intrathoracic pressure and, as a result, a lower cardiac output. The influence of arterial carbon dioxide pressure (PaCO_2_) on cerebral blood flow is significant. Hypocarbia is known to induce cerebral vasoconstriction, exacerbating cerebral ischemia, while hypercarbia is associated with increased cerebral blood flow, volume, and intracranial pressure [37,38]. Hyperventilation has been associated with increased hospital mortality and adverse neurological function [32,39,40,41,42,43,44,45,46,47]. Additionally, several studies suggest mild hypercarbia is correlated with reduced levels of neurological injury markers, along with improved cerebral oxygenation [34,48].

In terms of temperature management, hypothermia is a beneficial therapeutic approach for protecting the brain and other organs in patients who remain comatose—typically defined as an absence of meaningful response to verbal commands—after ROSC. However, there are still questions regarding the specific indications for hypothermia, the populations that may benefit, the optimal timing and duration of treatment, and the best methods for inducing, maintaining, and subsequently reversing hypothermia.

Neurological prognosis is difficult to deduce in the first 72 h, including in hypothermic patients [49]. Two randomized controlled trials reported that cooling comatose patients who experienced OHCA to a temperature range of 32 °C to 34 °C for 12 to 24 h, starting minutes to hours after ROSC, led to improved rates of neurologically intact survival at the time of hospital discharge [50,51]. Additional studies have also shown improved neurological outcomes following therapeutic hypothermia in initially comatose patients [52,53].

After the initial resuscitation, patients should undergo daily weaning trials with serial echocardiographic and hemodynamic assessments [54]. Patients with evidence of myocardial recovery can be considered for decannulation. If there is no evidence of myocardial recovery in the days preceding ECMO cannulation, durable ventricular assist device (VAD) and transplantation evaluations should be launched. Early discussions should be held with advanced referral centers if such interventions are not available at the patient’s institution.

## 5. Trends in Outcomes

The INCEPTION trial was one of the first large, multicenter clinical trials on eCPR, enrolling 160 patients across 10 centers who suffered OHCA with an initially shockable rhythm and no ROSC within 15 min. There was no significant difference in 30-day survival between the eCPR and conventional ACLS cohorts (20% vs. 16%, *p* = 0.52) [55]. The ARREST trial was conducted at the University of Minnesota with patients who presented with an OHCA and refractory ventricular fibrillation. The trial was stopped early as interim analysis revealed improved survival at hospital discharge in the eCPR group (43% vs. 7%) [56]. The PRAGUE study, a single-center trial, also did not find a difference in survival with neurologically favored outcomes at 180 days with the use of eCPR in OHCA [57]. Critics of the INCEPTION and PRAGUE trials point towards prolonged low-flow time (74 and 61 min), although mean time to cannulation was 59 min in the ARREST trial [58]. The INCEPTION trial also had longer cannulation times in comparison to ARREST (20 min vs. 7 min) and a higher incidence of unsuccessful cannulations (INCEPTION 11.5%, ARREST 0%, PRAGUE 2%). The CHEER trial was a single-center trial that included both eCPR for refractory OHCA and IHCA and therapeutic hypothermia (33 °C) for 24 h post-arrest. The CHEER trial had some of the lowest cannulation times, with a median time of 56 min and 54% hospital survival with full neurological recovery [59]. Furthermore, a recent meta-analysis suggested improved survival at discharge for IHCA eCPR utilization, though it found no difference in OHCA [60]. Despite the results being contradictory, these studies highlight that eCPR cannulation needs to be quick and efficient, which requires specialized training and adequate volume to maintain the necessary skills. To properly elucidate the importance of reduced transport and cannulation times, the Sub30 trial is currently taking place in London with the goal of delivering pre-hospital eCPR within 30 min of collapse [61]. A list of completed and ongoing eCPR trials is included (Table 1).

Additionally, adequate CPR prior to ECMO initiation is paramount for patient success. Guidelines by the AHA that were reviewed by the International Liaison Committee on Resuscitation (ILCOR) recommend the usage of quantitative waveform capnography and intra-arterial pressure measurements. Failure to achieve an end-tidal carbon dioxide (EtCO_2_) > 10 mmHg or diastolic pressure > 20 mmHg during initial resuscitation efforts is associated with poor survival and should be considered prior to ECMO placement [6,64].

A review of the ELSO database showed the median survival rate at hospital discharge of patients on ECMO after an OHCA was ~29% [3]. A recent multicenter retrospective analysis with 351 patients sheds some light on the prognosis of patients undergoing eCPR for OHCA. Along with the aforementioned risk factors, the study suggested that having cerebrovascular disease, high lactate (>14), or the lack of a shockable rhythm were risk factors for in-hospital mortality, while an age of 45–60 years, a higher pH (>7.065), and the use of an IABP were protective against mortality [65].

Further work is ongoing in OHCA and pre-hospital eCPR use. The largest study from Paris found pre-hospital eCPR was associated with improved survival at hospital discharge and neurological outcomes [66]. Additional work has been performed in the USA, Netherlands, Germany, and UK [5,67,68,69].

In terms of treating PCAS, Petermichl et al. showcased those individuals who had short, low, or no-flow times had better prognostic outcomes [5]. This can be credited to the fact that low ischemic times correlated with less infarcted tissue and a reduction in IRI. Markers of PCAS typically involve elevated lactate, S-100, and interleukin-6. S-100 is a cell marker seen in neuronal tissue, and leakage of this marker is correlated with poorer neurological outcomes. The study showed that S-100 is a reliable biomarker in determining neurological outcomes in patients that survived after eCPR [5].

A well-established marker for neurological injury is the measurement of neuron-specific enolase (NSE) levels in the blood post-eCPR. NSE is an enzyme involved in glucose metabolism within the cytoplasm of neurons found in both the central and peripheral nervous systems, as well as in neuroendocrine tissue, with an approximate biological half-life of 20 h [70]. Similarly to S-100, damage to neuronal tissue can result in leakage of this enzyme. A retrospective multicenter analysis aimed at elucidating the predictive power of NSE on neurological outcomes by measuring blood levels was conducted. There were no differences in NSE levels at 24 h post-arrest between the favorable and poor neurologic outcome groups; however, NSE levels were significantly higher at 48 (302 vs. 52 μg/L) and 72 (240 vs. 37 μg/L) hours in the poor neurologic group [71].

## 6. Complications Associated with eCPR

Initiation of eCPR is associated with several potential complications. In situations of prolonged CPR, injuries such as rib fractures, pulmonary contusion, blunt cardiac injury, intraabdominal injury, and pneumothorax are known to occur [72]. Due to the anticoagulation required at the time of cannulation and while on ECMO, bleeding is common. Previous work has reported an incidence of 11% intrathoracic bleeding, 6% intraperitoneal bleeding, and 1% hemopericardium due to a ruptured right ventricle [73]. Given the emergent nature of cannulation, vascular injury has also been reported to occur in approximately 18% of cases [72]. Following femoral cannulation, limb ischemia can develop due to the blockage of distal limb flow by the arterial cannula [74]. Evaluation of the distal limb for potential placement of a distal perfusion catheter (DPC) is critical to avoid potential amputation or death from prolonged limb ischemia [75]. Current literature indicates that the occurrence of limb ischemia is about 17% [76]. Various factors, including patients’ comorbid conditions, hemodynamic compromise, and size of the cannula, contribute to the overall risk of limb ischemia [77]. Further, while on ECMO, acute kidney injury requiring dialysis is common. A large database analysis from Taiwan found that survival at discharge was lower in patients requiring dialysis [78]. Following ROSC, the patient is also subject to secondary neurological injury from an imbalance between the delivery of oxygen to the brain and the brain’s metabolic demands for oxygen. This can arise from various factors such as reperfusion, dysfunction in the microcirculation, hyperoxia or hypoxia, hypercapnia or hypocapnia, low blood pressure, and elevated body temperature [79].

## 7. Cost Analysis

eCPR, being a resource-intensive intervention, comes with substantial costs. As the utilization of ECMO has increased, there have been various studies analyzing the multifactorial aspects of the cost associated with eCPR. In one study that utilized data from two ECMO centers in Australia, a Markov model of cost, quality of life, and survival outcomes was developed. The cost for survivors of eCPR was approximately $75,014 (95% confidence interval $66,209–$83,222), compared to an average cost of $117,000 for survivors after traditional ACLS [80].

Another potential way of looking at the cost-effectiveness of eCPR is by understanding the cost per quality-adjusted life year (QALY) saved. This value would allow for a standardization of the true value of utilizing eCPR for cases of OHCA. Although there have not been stringent cut-off values dictating an effective cost per QALY, some studies have suggested $50,000 per QALY as a threshold [81]. This threshold has been challenged in recent years, with some analyses suggesting $150,000 per QALY as a more reasonable cut-off [82]. A study that retrospectively analyzed all patients who underwent eCPR from 2013 to 2018 showed that eCPR achieved an average cost per QALY of $56,156, which falls well under the accepted parameters of cost-effectiveness. Furthermore, they showcased that the median operating cost per patient in the United States was approximately $125,683. These results, compared to other interventions such as dialysis, kidney transplantation, and orthotopic heart transplantation, showcased similar effectiveness as measured through cost per QALY [83].

A Japanese study showcased different costs for eCPR based on different pathologies of the OHCA. A systematic review of 120 patients who underwent eCPR showcased a stark difference in cost associated with ventricular fibrillation or pulseless ventricular tachycardia (VF/VT; *n* = 59) versus asystole or pulseless electric activity (ASY/PEA; *n* = 61). The total cost associated with patients presenting with ASY/PEA was almost double that of patients presenting with VF/VT. This could either be credited to the increase in post-arrest care time, ECMO intervention, or increased personnel demand. Furthermore, the calculated cost per QALY was about $29,447 for patients with ASY/PEA and $11,081 for patients with VF/VT undergoing eCPR [84]. The differences in etiology for OHCA have hence been suggested to dictate decision-making when placing a patient on ECMO. These factors, along with contributing to the general success rate of the intervention, also further influence the cost-effectiveness assessment of the intervention. The quantitative differences in cost/cost per QALY could be credited to the year they were implemented and the hospital system/country they were affiliated with. Moreover, additional cost analysis based on different etiologies/hospital systems and demographic groups needs to be undertaken to further understand the cost-effectiveness of eCPR.

## 8. Ethical Considerations

Due to the lack of strong guidelines surrounding the use of eCPR, the ethical implications of using ECMO in times of emergency have not been delineated. It is generally agreed upon that it would be unethical to utilize ECMO to supplement resuscitation efforts if a patient is felt to not benefit from eCPR. Furthermore, the inability to obtain explicit consent from patients who are indicated for eCPR further poses a challenge to the ethical limitation of this intervention [85]. For those patients who are successfully cannulated but are not candidates for advanced therapies (revascularization, transplant, durable ventricular assist device), these patients are effectively stuck on ECMO.

A study on eCPR indicated better survival rates for patients compared to traditional CPR but also associated eCPR with a higher incidence of comatose and vegetative states [86]. This could potentially be seen as worse than death by patients [87]. This further leads to ethical complications regarding how different countries view brain death and its implications on organ donation [85]. Those patients undergoing eCPR but subsequently dying are found to have longer intervals from admission to organ procurement. This has been shown to have a significant impact on lung graft survival rates [88]. Although contraindications for an intervention should never be considered based on prospective donor outcomes, their implications have been documented.

A single-center analysis of organ procurement post-OHCA yielded data regarding the relationship between low flow times and consequent organ procurement. Among 307 adults with OHCA who received eCPR (with a median low-flow time of 70 min), 256 individuals (83%) died during their hospital stay. Organ donation of at least one solid organ was recorded in 58 (19%) patients, resulting in a total of 167 solid organs. The rate of solid organ donation dropped from 19% to 16% for patients with a low-flow time of less than 60 min and further decreased to 11% for those with a low-flow time of less than 60 min accompanied by an initial shockable rhythm. In total, 196 individuals (comprising 29 survivors with a favorable neurological outcome and 167 potential recipients of at least one solid organ) were positively impacted by eCPR [89].

## 9. Conclusions

With the advancements of mechanical circulatory support technologies, extracorporeal resuscitation techniques for cardiac arrest in and outside the hospital have continued to evolve. The lack of strong international or regional guidelines has resulted in a varied amount of data related to the outcomes of eCPR. Outcomes thus far suggest a potential benefit in the early initiation of eCPR, though further work is needed to properly identify patients in whom this intervention would have the most impact.

## Figures and Tables

**Table 1:** List of completed and ongoing eCPR trials.

Study	Region	Age	Presentation	Intervention	Time to Cannulation (minutes)	Sample Size	Hospital Survival
ARREST [56]	Single-center, University of Minnesota, USA	18–75	OHCA, VF or VT, no ROSC after 3 shocks, transfer time < 30 min	ACLS	N/A	15	1 (7%)
				eCPR	59 (SD: 28)	15	6 (43%)
PRAGUE [57]	Single-center, Charles University, Prague, Czech Republic	18–65	Witnessed OHCA, >5 min ACLS	ACLS	N/A	132	29 (22%)
				eCPR	61 (IQR: 55–70)	124	39 (32%)
INCEPTION [55]	Multicenter, The Netherlands	18–70	Witnessed VT/VF OHCA, refractory (>15 min ACLS)	ACLS	N/A	64	13 (20%)
				eCPR	74 (IQR: 63–87)	70	14 (20%)
CHEER [59]	Single-center, The Alfred Hospital, Australia	18–65	VF IHCA or OHCA, CPR within 10 min of arrest	eCPR	56 (IQR: 40–85)	26	14 (54%)
Sub30 [61]	Multicenter, London	18–65	Witnessed OHCA, CPR within 3 min of arrest, refractory arrest > 20 min	eCPR	Goal < 30 min after arrest	Ongoing	Ongoing
EROCA [62]	Single-center, The University of Michigan, USA	18–70	OHCA, initial shockable rhythm, transfer time of <30 min	eCPR	66.2 (SD: 16.7)	15 (5 qualified for eCPR)	0 (0%)
ON-SCENE [63]	Multicenter, Netherlands	18–50	Witnessed VT/VF OHCA, refractory arrest > 20 min	eCPR	Ongoing	Ongoing	Ongoing

ACLS, advanced cardiac life support; AED, automated external defibrillator; eCPR, extracorporeal cardiopulmonary resuscitation; OHCA, out-of-hospital cardiac arrest; IHCA, in-hospital cardiac arrest VF, ventricular fibrillation; VT, ventricular tachycardia.
